# Peritumoral tissue (PTT): increasing need for naming convention

**DOI:** 10.1038/s41416-024-02828-y

**Published:** 2024-09-02

**Authors:** Dzenis Koca, Behnoush Abedi-Ardekani, Joel LeMaoult, Laurent Guyon

**Affiliations:** 1 Interdisciplinary Research Institute of Grenoble, IRIG-Biosanté, University Grenoble Alpes, CEA, INSERM, UMR 1292, F-38000 Grenoble, France; 2 International Agency for Research on Cancer (IARC/WHO), Genomic Epidemiology Branch, Lyon, France; 3 Commissariat à l’Energie Atomique et aux Energies Alternatives, DRF, Francois Jacob Institute of Biology, Hemato-Immunology Research Department, Saint-Louis Hospital, Paris, France; 4 INSERM U976 HIPI Unit, IRSL, Université Paris, Paris, France

**Keywords:** Cancer genomics, Tumour biomarkers, Surgical oncology, Cancer genomics

## Abstract

Various terms are used to describe non-malignant tissue located in the proximity of a tumor, belonging to the organ from which the tumor originated. Traditionally, these tissues, sometimes called “normal adjacent tissue” have been used as controls in cancer studies, and were considered representative of morphologically healthy, non-cancerous tissue. However, with the advancement of OMIC technologies, such tissues are increasingly recognized to be distinct from both tumor and healthy tissues. Furthermore, properties, characteristics, and role of these tissues in cancer formation and progression is increasingly studied. In order to make future research in this area more harmonized and more accessible, as well as to counter the widespread perception of normalcy, we are advocating the need for standardized naming convention. For this purpose, we propose the use of neutral and comprehensive term “Peritumoral Tissue” along with the acronym “PTT”. While significant amount of data on these tissues are publicly available, reuse of such data remains limited due to a lack of information on sample collection procedures. In order to facilitate future reuse of the data, we suggest a list of features that should be documented during sample collection procedures. These recommendations can aid the definition of Standard Operating Procedures.

## Introduction

Cancer accounts for almost 20% of premature death worldwide [1]. Peritumoral tissues (PTT), which are non-tumor tissues located in close proximity to a tumor and originate from the same organ, are increasingly recognized to harbor complementary information on early tumorigenesis, metastasis, recurrence, and treatment response, as well as prognosis [2–6]. While most cancer studies focus on cancer itself, this growing body of research suggests the importance of also investigating PTT. To emphasize that PTT differs from healthy tissue (referring to tissue from individuals unaffected by cancer), and to facilitate scientific communication and knowledge dissemination, we draw attention to the growing need for an appropriate naming convention. Additionally, we will highlight key information about the peritumoral tissue sampling procedure that can enhance the precision and informativeness of bioinformatics analysis.

Advancements in molecular biology and sequencing technologies, along with reduced costs and increased accessibility, have revolutionized cancer research. These approaches have resulted in a more profound understanding of cancer pathology, as well as molecular abnormalities that lead to cancer formation, sustenance, progression, and metastasis. In many studies, tumors are analyzed alongside PTT, where PTT serves as a control or baseline [7, 8]. Due to the absence of macroscopic^1^ and microscopic^2^ signs of malignancy, PTT is considered to adequately represent morphologically healthy, non-tumoral tissue and is often used as a “healthy” control [5, 7]. Furthermore, the use of paired tumor-PTT samples is often justified due to the experimental advantages it brings. First, the use of paired tumor-PTT samples reduces interpatient genetic variability, as both tumor and PTT samples originate from the same patient. The second advantage is decreased anatomical variability as PTT samples are taken from the same tissue type from which the tumor originated. This is usually the case for proteomic, transcriptomic, and epigenetic studies (methylation and genome-wide associations), as for genomic studies, DNA is often isolated from peripheral blood leukocytes. The third advantage is that PTT samples are more accessible than healthy tissue samples from non-cancer patients, especially in cases where radical surgeries (partial or total organ removal) are necessary steps in cancer treatment.

While most studies operate under the assumption that PTT is representative of healthy tissue, a growing amount of evidence suggests that this is not the case. The concept that PTT is “healthy” was first debated by Slaughter et al. in 1953 [9]. Namely, the authors propose that the high rate of recurrence of Oral Squamous Cell Carcinoma originates from the fact that PTT has been preconditioned by the same carcinogenic event that led to the rise of tumors in the first place, leading to the formation of “field cancerization”. Since this seminal paper, genetic and epigenetic abnormalities that lead to the formation of cancerized fields have been described in various tumors and pre-malignant diseases [5, 8, 10]. In 2017, Aran and colleagues conducted a pan-cancer study, comparing the transcriptomic profiles of primary tumors, corresponding PTT (from the TCGA database), and healthy tissues from autopsies (from the GTEx database) of eight tumor types [7]. They found that PTT is transcriptionally different from both primary tumor tissues and healthy tissues and is instead found in the transcriptional middle ground. Similar results, showing that PTT is transcriptionally distinct from healthy tissues of corresponding organs, have been reported by other groups as well [6, 11–15]. Aran and colleagues suggested that the distinct transcriptome of PTT could arise from the process of field cancerization, as well as from the cancer-induced inflammation and putative signals that originate from the tumor with the latter being the most probable source [7].

Additionally, the authors show that increased expression of a tumor-adjacent-specific signature can be detected up to 4 cm from the tumor (maximum distance in the dataset), with a tendency for gradual decrease after 2 cm [7]. Other groups, as reviewed by Gadaleta et al., have also shown that genomic instability, telomere content, allelic imbalance, and transcriptomic aberrations decrease as a function of distance from the tumor [8]. On the other hand, a recent study that investigated the impact of the distance of the transcriptomic subtype of PTT of breast cancer patients showed no clear distinction in PTT subtype based on the distance from the tumor [16]. Overall, the impact of distance from the tumor on peritumoral tissue, as well as the underlying cause, is still elusive, and more research is needed.

Furthermore, an increasing body of evidence suggests that PTT could be useful in understanding early mutational events in tumorigenesis, as well as understanding the impact of tumors on surrounding tissue, angiogenesis, and even serve as a source of prognostic and diagnostic biomarkers [2–8, 10, 16, 17].

## Need for naming convention

As presented, increasing evidence shows that PTT is not entirely representative of healthy tissue. However, the term “normal adjacent tissue” (“NAT”), and various permutations thereof, are still commonly used to denote PTT (Supplementary Fig. 1, Supplementary Table 1). While one can argue that PTT is often confirmed to be “histologically normal”, a growing body of evidence is suggesting that histological normalcy does not always imply molecular normalcy [4, 7, 11]. Therefore, the term “normal adjacent tissue” can lead to misconception that PTT is normal, and in turn to misinterpretation of results. Subsequently, some key information can be missed due to this misconception.

In addition to the term “normal adjacent tissue”, other terms, such as ones presented in Table 1 are interchangeably used in literature. It is important to note that terminology can be context-dependent, and can vary across different disciplines and research areas, as well as across organs affected by tumor. However, it seems that such variations are not always based on standard medical/histological definitions and are prone to be used based on personal interpretations. There is a variation among terminologies used by different researchers. For example, researchers commonly use the term ‘mucosa’ to address the entire wall thickness, which is not correct. In standard medical and histological terminology, mucosa applies to the first layer of the entire wall thickness in some organs such as the gastrointestinal or the lower urinary tract, while such specific layering is not part of the histological structure of most of the organs such as breast, pancreas, kidney, etc. Although using the term ‘mucosa’ in some organs (such as gastrointestinal tract) could be correct, it is prone to lead to misinterpretation or misunderstanding, and can even be inappropriately applied to the areas where such a structure does not exist. On the other hand, this term an be appropriate if the tissue is collected by some specific procedures, such as endoscopy, in which the procedure of punch biopsy by endoscopy does not allow to penetrate deeper than mucosa. Although such precisions might be respected by some registries during sample collection and addressing the method of sample collection could help to clarify the depth of wall thickness, we recommend using a more general and less confusing terminology such as ‘tissue’ when registering the collected sample. Another example of terminology misuse is that the terms “adjacent non-neoplastic tissue” or “benign adjacent tissue” are often used when histological normalcy is not implied, particularly in studies that investigate immune infiltration and cell composition of PTT. All things considered, such jargon poorly transfers between fields, is not standardized, and limits the use of “keywords” for literature discovery [18]. Additional problems can arise when one of the terms used to describe PTT has different meanings between different fields. For example, the term “tumor macroenvironment” is sometimes used to denote PTT, while in research on systemic effects of cancer (such as cachexia and paraneoplastic syndrome) “tumor macroenvironment” is used to describe distant organs and even the whole human body [19]. Such diversity of names and lack of clear naming convention makes it harder to discover and understand adequate literature, and particularly limits usage of keywords in search engines. To the best of our knowledge, various names used to denote PTT are not incorporated into “The Medical Subject Headings (MeSH)” thesaurus [20]. Additionally, poor transfer of meaning across disciplines can obscure “the big picture” and confuse readers, especially if a reader is new to the field. On the other hand, common names can in turn bring the community closer, and spark new interest in research particularly oriented towards investigation of peritumoral tissues.

**Table 1:** Terms and their abbreviations that are frequently used to denote peritumoral tissue.

Terms often used to denote peritumoral tissue	Abbreviations encountered in literature
Normal adjacent (tissue) Normal tissue adjacent to tumor/cancer Adjacent normal cells Normal appearing adjacent to tumor Tumor-adjacent normal Cancer-adjacent normal	NAT/AN/NA/TAN
Peritumoral tissue Peritumor Peritumoral stroma	PT/PTT
Healthy adjacent tissue	HA/AH
Tumor macroenvironment	–
Benign adjacent tissue Non-malignant adjacent tissue	–
Tumor-adjacent histologically normal Histologically normal adjacent tissues Histologically normal tissue Histologically normal cancer-adjacent	TAHN/HN/HNCA
Cancer-adjacent tissue	CA/CAT
Tumor-adjacent tissue, Tumor-adjacent area, Adjacent to tumor	TA/TAT
Extratumoral microenvironment Peritumor microenvironment	
Normal-appearing tissue Normal tissue	–
Morphologically normal tissue	–
Non-pathological tissue adjacent to tumor Non-tumor tissue, Non-cancer tissue	–
Adjacent non-neoplastic	–

Terms are not ordered in any way.

Given the rapid developments in the field of cancer research and application of more and more sophisticated technologies that require precise data, standardization of terminology becomes a necessary element to avoid confusion and misinterpretation. This is an important element to be considered while creating Standard Operating Procedures (SOPs). With the aim of harmonization of future literature, and in order to counter the misconception of “normalcy”, we are encouraging use of a common and neutral term “peritumoral tissue” and the acronym “PTT,” when referring to tissues that are found in proximity to the tumor, and are of same anatomical origin as a tumor.

## Rich metadata on PTT improves reusability of data

**Fig. 1:**
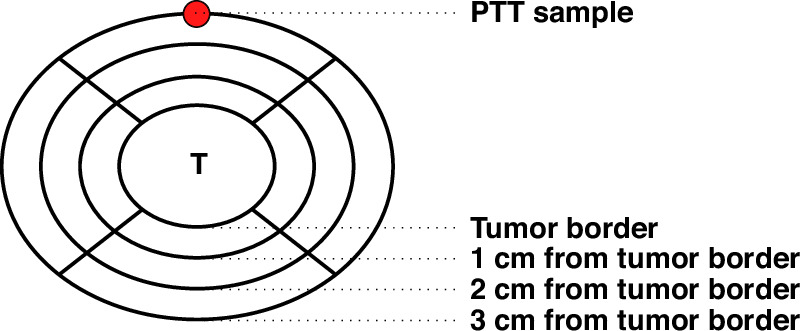
Graphical representation of the hypothetical PTT sample. Sample is 1 cm^3^ in size, taken 3 cm from superior from the tumor border. Tumor is oval, with longer axis set in transverse (horizontal) plane.

With the establishment of FAIR data principles, and to facilitate reuse of data and research reproducibility, authors are encouraged to share data obtained from an experiment [21]. Often, data on tumor tissue samples are well annotated and clinical data of patients are made available; however, this is not the case for metadata of PTT. As mentioned earlier, PTT is often used as a control in cancer studies, and as a result, metadata on PTT samples are often not recorded and remain incomplete. For example, the distance from the tumor at which each PTT sample is taken is rarely specified. The impact of the distance on molecular and transcriptional aberrations remain unclear. Some previous studies have suggested ‘safe’ distances from a diseased or malignant tissue based on the ‘back to normal’ function of the organ and have reported it to be as short as 5 mm in kidney [22]. Khemees et al. reported renal glomerular viability improving from 58% to 92% in 2.5 mm and 10 mm from tumor, respectively [23]. If molecular features also follow such clinical improvements in distance from tumor is not clear [7]. It is also possible that the ‘safe’ distance from a disease status is organ dependent and variable, and is based on the morphology and function of each organ. Supporing this notion is the fact that in even in routine practice of pathology there is not a unique distance from surgical resection margin of a malignant lesion which is considered as safe margin. Instead required distance for surgical recection margine is organ dependent. Additionally, based on the tumor size and the method of surgery (partial versus total resection), providing a fixed distance from tumor as a guideline is not feasible. As a result, we strongly recommend collecting PTT as far as possible from a tumoral lesion and emphasize to record the distance. The inclusion of such information in publicly available datasets can facilitate research on this topic and improve the precision of guidelines on PTT sampling. Furthermore, such information can also be used as a confounding variable during bioinformatics analysis. We have included some of the important information about PTT samples in Table 2 as well as graphical representation of such variables in Fig. 1. We are well aware that tumors can have irregular shapes and orientations, and that most of the mentioned information can be hard or impossible to capture correctly. Nevertheless, capturing as much information as possible can prove useful during downstream data analysis and can be essential when data is reused later on.

**Table 2:** Important characteristics of PTT, which can facilitate the bioinformatics analysis and reuse of data.

Characteristic	Definition	Importance
Sampling distance	Distance from the tumor border at which PTT sampling was performed.	Current data indicates that PTT gene expression, as well as molecular aberrations, could be influenced by this distance.
Anatomical location (topography)	Information on PTT sampling location in the reference to a tumor, organ, and exact topography in which tumor is found.	This information can help remove unwanted sources of variation. Additionally, this information is particularly important when more than one sample of PTT is taken.
Gross morphology and shape of tumor	Whether the tumor is fungating, ulcerative, infiltrative, depressed, etc.	This information can help to explain how precise PTT sampling is, particularly for tumors with vague gross morphology such as extremely fibrotic ones.
Tumor border clarity	Whether tumor is infiltrative, or well demarcated and clearly discerned.	In cases of infiltrative tumors, tumor borders are hard to discern, and precise measurement of sampling distance is hard to be recorded.
Sample size	Dimensions of PTT sample (LxWxH).	Size of PTT sample can influence sampling resolution.
Tumors with specific features such as cystic components, hemorrhage, massive necrosis, etc.	Examples: mixed cystic and solid structures; cystic change with hemorrhage; massive necrosis challenging the collection of tissue from non-necrotic areas; papillary structures; marked calcifications; scirrhous wall with unclear tumor border.	Some tumors show cystic degenerations or are cystic in nature. Defining the location of PTT sampling or measuring the distance is difficult. In such situations, documentation of the structure by adding schemes or even photos in the data collection registries are very useful and avoid misinterpretations.
PTT tissue with specific morphologies.	In case PTT is taken from a specific lesion.	In cases where PTT is not histologically normal and is taken from any lesion or pathology, such as a suspicious pre-malignant lesion.
Presence of multiple tumors	Whether there are multiple primary malignancies.	Presence of multiple tumors or synchronous malignancies are special cases in which role played by PTT is of particular interest. In this situation, the location of collected PTT and distance from each tumor should be recorded separately.

It applies and refers to a standard classification system, such as ICD-O to define topographies in a standard way. The key element is to document all observations (such as un-clarity of the tumor border) precisely.

## Conclusion

Peritumoral tissues (PTT) are increasingly a topic of scientific research, offering complementary information on early steps of tumorigenesis, as well as mechanisms of metastasis, progression, and recurrence of cancer. While investigating literature on PTT, we have identified two major issues that could hinder PTT-oriented research: variable and often imprecise nomenclature and insufficiently characterized data. The proposed standardization of terminology aims to improve how we communicate and understand research in which peritumoral tissue is used, especially studies where investigation of properties of PTT is the primary goal. Structured and precise terminology can improve the dissemination of information and in turn can lead to better understanding and critical assessment of results. Additionally, standardized terminology can facilitate the sharing and reuse of data and can prevent the loss of valuable information. An important goal of standardization is to streamline future research and point to the role that PTT plays in some of the key steps of tumor pathology. It is important to note that search engines, such as PubMed or Google Scholar, are valuable scientific tools, and a lack of consistent terminology can hinder the discovery of adequate literature. We provide as Supplementary Material a link to search publications with at least one of the terms in abstract through these search engines to ease the identification of relevant associated works (Supplementary Table 1).

While PTT is often used as a control in cancer studies, and a significant amount of data is publicly available, the reusability of such data is limited due to insufficient metadata. The lack of data regarding the sampling distance from the tumor has already been reported as a limitation in several retrospective studies [3, 7]. Additionally, recent work by Lau et al. suggests that other information, such as anatomical orientation and tumor heterogeneity, can also prove to be useful [16]. The collection of samples and metadata, as well as the downstream analysis of samples (including bioinformatics analysis of resulting data), is a multi-disciplinary effort. Success in multi-omics studies require control of pre-analytical, analytical, and post-analytical elements, an effort that becomes more specialized and needs the application of multidisciplinary approaches. Experts in each field should work in teams and be involved from the study design to data analyses. As for the matter of sample and metadata collection, the opinion of a bioinformatician on potential confounding variables, or the expertise of a pathologist on sample collection, annotation and inclusion, can greatly reduce issues encountered during downstream data analysis and increase the reusability of the data themselves.

## Supplementary information

Supplementary Figure 1

Supplementary Table 1

## Footnotes

1 Referring to peritumoral tissue samples that are taken at substantial distance from tumor border.

2 Referring to practice where, in some studies, pathologists investigate peritumoral tissue for presence of malignant cells.
