# Supplementary material for: Peritumoral tissue (PTT): increasing need for naming convention
Source: Br J Cancer. 2024 Sep 2;131(7):1111–5. doi: 10.1038/s41416-024-02828-y (PMC11443153; doi:10.1038/s41416-024-02828-y)

Supplementary Figure 1: Comparison of number of articles that either contain terms Peritumoral tissue or Normal-adjacent tissue, or terms that imply or do not imply normalcy in title and/or abstract. A) Venn diagram showing number of overlapping results depending on the queries described in Supp.Table 1; B) Number of results between years 2000 and 2023 in which, when describing PTT, authors use terms that imply normalcy(in Red) or do not imply normalcy(in Blue); C) Number of results between years 2000 and 2023 in which, when describing PTT, authors use term “Normal adjacent tissue”(in Red, similar terms included) or term “Peritumoral tissue” (in Blue). Results retrieved from PubMed search engine, while using queries described in Supp. Table 1.

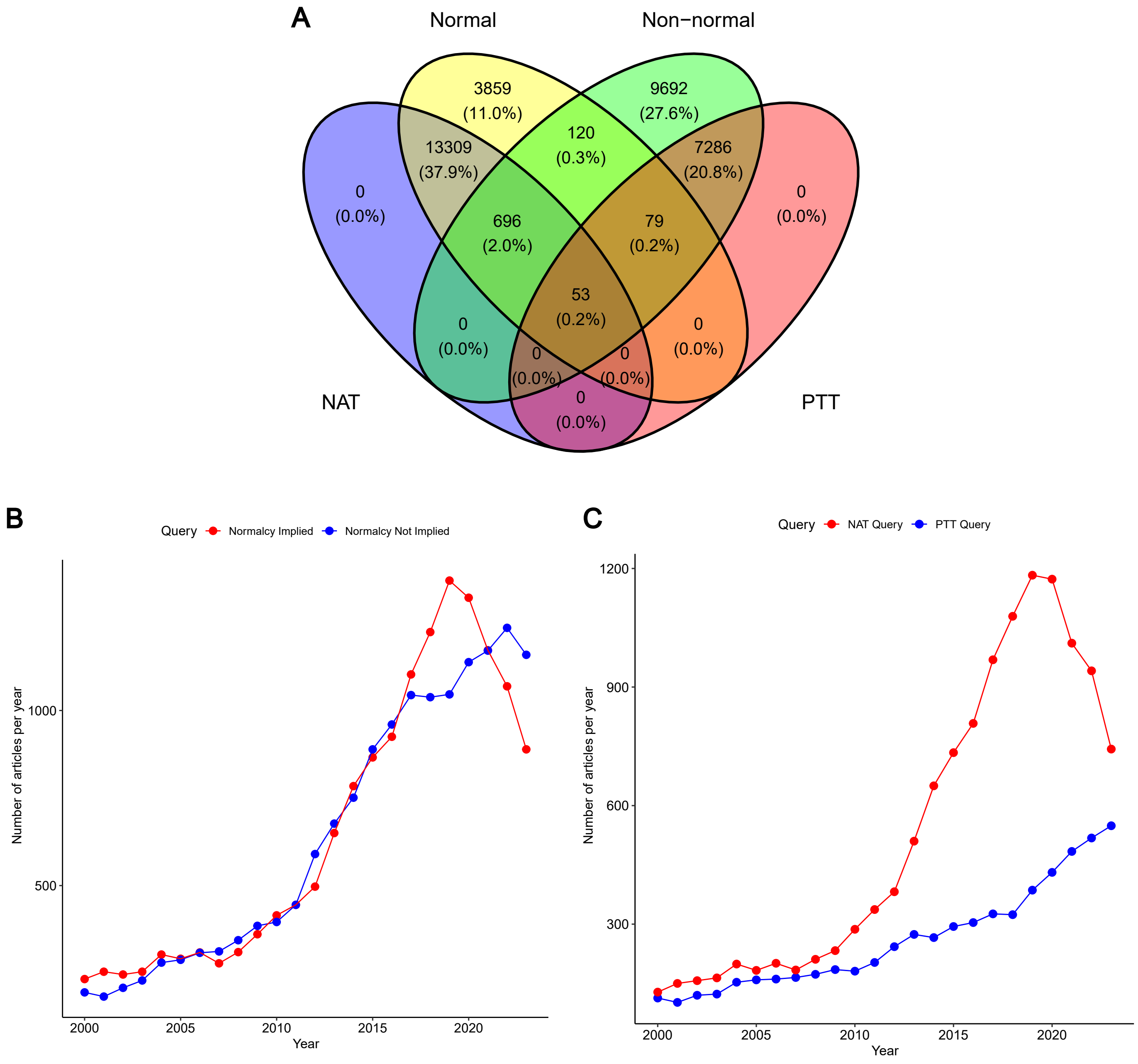

Supplement: Supplementary file 1 — Supplementary Figure 1 [file 41416_2024_2828_MOESM1_ESM.pdf]
