# Supplementary material for: Peritumoral tissue (PTT): increasing need for naming convention
Source: Br J Cancer. 2024 Sep 2;131(7):1111–5. doi: 10.1038/s41416-024-02828-y (PMC11443153; doi:10.1038/s41416-024-02828-y)
Supplement: Supplementary file 2 — Supplementary Table 1 [file 41416_2024_2828_MOESM2_ESM.docx]

**Supplementary Table 1**: Pubmed and Google scholar query hyperlinks links used to search for articles containing some of the most often seen names for peritumoral tissue. PubMed queries show results in articles containing these terms in Title or Abstract. Both services were accessed on 30-May-2024.

| Terms | | Search engine | **Link** | Number of results |
| --- | --- | --- | --- | --- |
| - Peritumoral Tissue (PTT) | | PubMed | [**PTT query**](https://pubmed.ncbi.nlm.nih.gov/?term=(%22Peritumor%22%5bTitle/Abstract%5d%20OR%20%22Peritumoral%22%5bTitle/Abstract%5d)%20AND%20(%22Tumor%22%5bTitle/Abstract%5d%20OR%20%22Cancer%22%5bTitle/Abstract%5d%20OR%20%22Carcinoma%22%5bTitle/Abstract%5d%20OR%20%22Adenoma%22%5bTitle/Abstract%5d%20OR%20%22Sarcoma%22%5bTitle/Abstract%5d))**^a^** | 7418 |
|  |  | Google Scholar | [**PTT query**](https://scholar.google.com/scholar?q=%22Peritumor%22+OR+%22Peritumoral+tissue%22) | 14700^b^ |
| - Normal Adjacent Tissue (NAT) | | PubMed | [**NAT query**](https://pubmed.ncbi.nlm.nih.gov/?term=%28%22Normal+adjacent%22%5BTitle%2FAbstract%5D+OR++%22Adjacent+normal+%22%5BTitle%2FAbstract%5D+OR++%22Neighboring+Normal%22%5BTitle%2FAbstract%5D+OR++%22Proximal+Normal%22%5BTitle%2FAbstract%5D+OR++%22Normal+tissue+adjacent%22%5BTitle%2FAbstract%5D+OR+%22Surrounding+Normal+Tissue%22%5BTitle%2FAbstract%5D%29+AND+%28%22Tumor%22%5BTitle%2FAbstract%5D+OR++%22Cancer%22%5BTitle%2FAbstract%5D+OR++%22Carcinoma%22%5BTitle%2FAbstract%5D+OR++%22Adenoma%22%5BTitle%2FAbstract%5D+OR++%22Sarcoma%22%5BTitle%2FAbstract%5D%29&show_snippets=off&sort=date&size=200)**^a^** | 14058 |
|  |  | Google Scholar | [**NAT query**](https://scholar.google.com/scholar?hl=fr&as_sdt=0%2C5&q=%28%22Normal+adjacent%22+OR++%22Adjacent+normal%22+OR++%22Neighboring+Normal%22+OR++%22Proximal+Normal%22+OR++%22Normal+tissue+adjacent%22OR+%22Surrounding+Normal+Tissue%22%29+AND+%28%22Tumor%22+OR++%22Cancer%22+OR++%22Carcinoma%22+OR++%22Adenoma%22+OR++%22Sarcoma%22%29&btnG=) | 16900^b^ |
| Terms that imply normalcy | - Normal Adjacent Tissue - Surrounding Normal Tissue - Normal-appearing Tissue - Histologically Normal Tissue - Morphologically Normal Tissue - Healthy Adjacent Tissue - Adjacent Benign Tissue | PubMed | [**“Normalcy implied” query**](https://pubmed.ncbi.nlm.nih.gov/?term=%28%22Normal+adjacent%22%5BTitle%2FAbstract%5D+OR++%22Adjacent+normal+%22%5BTitle%2FAbstract%5D+OR++%22Neighboring+Normal%22%5BTitle%2FAbstract%5D+OR++%22Proximal+Normal%22%5BTitle%2FAbstract%5D+OR++%22Normal+tissue+adjacent%22%5BTitle%2FAbstract%5D+OR+%22Surrounding+Normal+Tissue%22%5BTitle%2FAbstract%5D+OR++%22Normal+appearing%22%5BTitle%2FAbstract%5D+OR+%22Histologically+Normal%22%5BTitle%2FAbstract%5D+OR+%22Healthy+Adjacent%22%5BTitle%2FAbstract%5D+OR+%22Adjacent+Healthy+%22%5BTitle%2FAbstract%5D+OR+%22Benign+Adjacent+%22%5BTitle%2FAbstract%5D+OR+%22Morphologically+normal+%22%5BTitle%2FAbstract%5D+OR+%22Adjacent+Benign+%22%5BTitle%2FAbstract%5D%29+AND++%28%22Tumor%22%5BTitle%2FAbstract%5D+OR++%22Cancer%22%5BTitle%2FAbstract%5D+OR++%22Carcinoma%22%5BTitle%2FAbstract%5D+OR++%22Adenoma%22%5BTitle%2FAbstract%5D+OR++%22Sarcoma%22%5BTitle%2FAbstract%5D%29&show_snippets=off&sort=date&size=200)**^a^** | 18116 |
|  |  | Google Scholar | [**“Normalcy implied” query**](https://scholar.google.com/scholar?hl=fr&as_sdt=0%2C5&q=%28%22Normal+adjacent%22+OR++%22Adjacent+normal+%22+OR++%22Neighboring+Normal%22+OR++%22Proximal+Normal%22+OR++%22Normal+tissue+adjacent%22+OR+%22Surrounding+Normal+Tissue%22+OR++%22Normal+appearing%22+OR+%22Histologically+Normal%22+OR+%22Healthy+Adjacent%22+OR+%22Adjacent+Healthy+%22+OR+%22Benign+Adjacent+%22+OR+%22Morphologically+normal+%22+OR+%22Adjacent+Benign+%22%29+AND++%28%22Tumor%22+OR++%22Cancer%22+OR++%22Carcinoma%22+OR++%22Adenoma%22+OR++%22Sarcoma%22%29+AND+%22Tissue%22&btnG=)**^b^** | 18600 |
| Terms that do not imply normalcy | - Peritumoral Tissue - Juxtatumoral Tissue - Non-malignant (non-neoplastic) Adjacent Tissue - Non-tumor tissue - Tumor-adjacent tissue - Tumor-surrounding tissue - Cancer-adjacent tissue - Tumor Macroenvironment - Extratumoral microenvironment | PubMed | [**“Normalcy not implied” query**](https://pubmed.ncbi.nlm.nih.gov/?term=%28%22Peritumor%22%5BTitle%2FAbstract%5D+OR+%22Peritumoral%22%5BTitle%2FAbstract%5D+OR++%22juxtatumoral%22%5BTitle%2FAbstract%5D+OR++%22Non+malignant+Adjacent%22%5BTitle%2FAbstract%5D+OR++%22Tumor+Adjacent%22%5BTitle%2FAbstract%5D+OR+%22Tumor+macroenvironment%22%5BTitle%2FAbstract%5D+OR+%22Adjacent+non-neoplastic%22%5BTitle%2FAbstract%5D+OR+%22Non-tumor%22%5BTitle%2FAbstract%5D+OR+%22Cancer-adjacent%22%5BTitle%2FAbstract%5D+OR+%22Adjacent+to+Tumor%22%5BTitle%2FAbstract%5D+OR+%22Tumor-Surrounding%22%5BTitle%2FAbstract%5D+OR+%22Extratumoral+microenvironment%22%5BTitle%2FAbstract%5D%29+AND++%28%22Tumor%22%5BTitle%2FAbstract%5D+OR++%22Cancer%22%5BTitle%2FAbstract%5D+OR++%22Carcinoma%22%5BTitle%2FAbstract%5D+OR++%22Adenoma%22%5BTitle%2FAbstract%5D+OR++%22Sarcoma%22%5BTitle%2FAbstract%5D%29&show_snippets=off&sort=date&size=200)**^a^** | 17926 |
|  |  | Google Scholar | [**“ Normalcy not implied” query**](https://scholar.google.com/scholar?hl=fr&as_sdt=0%2C5&q=%28%22Peritumor%22+OR+%22Peritumoral%22+OR++%22juxtatumoral%22+OR++%22Non+malignant+Adjacent%22+OR++%22Tumor+Adjacent%22+OR+%22Tumor+macroenvironment%22+OR+%22Adjacent+non-neoplastic%22+OR+%22Cancer-adjacent%22+OR+%22Adjacent+to+Tumor%22+OR+%22Tumor-Surrounding%22+OR+%22Extratumoral+microenvironment%22%29+AND++%28%22Tumor%22+OR++%22Cancer%22+OR++%22Carcinoma%22+OR++%22Adenoma%22+OR++%22Sarcoma%22%29+AND+%22tissue%22+and+-%22Normal%22&btnG=)**^b^** | 23600 |
| - FULL query | - Query using all terms above | PubMed | [**FULL query**](https://pubmed.ncbi.nlm.nih.gov/?term=%28%22Peritumor%22%5BTitle%2FAbstract%5D+OR+%22Peritumoral%22%5BTitle%2FAbstract%5D+OR++%22juxtatumoral%22%5BTitle%2FAbstract%5D+OR++%22Non+malignant+Adjacent%22%5BTitle%2FAbstract%5D+OR++%22Tumor+Adjacent%22%5BTitle%2FAbstract%5D+OR+%22Tumor+macroenvironment%22%5BTitle%2FAbstract%5D+OR+%E2%80%9CExtratumoral+microenvironment%E2%80%9D%5BTitle%2FAbstract%5D+OR+%22Adjacent+non-neoplastic%22%5BTitle%2FAbstract%5D+OR+%22Non-tumor%22%5BTitle%2FAbstract%5D+OR+%22Cancer-adjacent%22%5BTitle%2FAbstract%5D+OR+%22Adjacent+to+Tumor%22%5BTitle%2FAbstract%5D+OR+%22Tumor-Surrounding%22%5BTitle%2FAbstract%5D+OR+%22Extratumoral+microenvironment%22%5BTitle%2FAbstract%5D+OR+%22Normal+adjacent%22%5BTitle%2FAbstract%5D+OR++%22Adjacent+normal+%22%5BTitle%2FAbstract%5D+OR++%22Neighboring+Normal%22%5BTitle%2FAbstract%5D+OR++%22Proximal+Normal%22%5BTitle%2FAbstract%5D+OR++%22Normal+tissue+adjacent%22%5BTitle%2FAbstract%5D+OR+%22Surrounding+Normal+Tissue%22%5BTitle%2FAbstract%5D+OR++%22Normal+appearing%22%5BTitle%2FAbstract%5D+OR+%22Histologically+Normal%22%5BTitle%2FAbstract%5D+OR+%22Morphologically+normal+tissue%22%5BTitle%2FAbstract%5D+OR+%22Healthy+Adjacent%22%5BTitle%2FAbstract%5D+OR+%22Adjacent+Healthy+%22%5BTitle%2FAbstract%5D+OR+%22Benign+Adjacent+%22%5BTitle%2FAbstract%5D+OR+%22Morphologically+normal+%22%5BTitle%2FAbstract%5D+OR+%22Adjacent+Benign+%22%5BTitle%2FAbstract%5D%29+AND+%28%22Tumor%22%5BTitle%2FAbstract%5D+OR++%22Cancer%22%5BTitle%2FAbstract%5D+OR++%22Carcinoma%22%5BTitle%2FAbstract%5D+OR++%22Adenoma%22%5BTitle%2FAbstract%5D+OR++%22Sarcoma%22%5BTitle%2FAbstract%5D%29&show_snippets=off&sort=date&size=200) | 35095 |
|  |  | Google Scholar | [**FULL query**](https://scholar.google.com/scholar?hl=fr&as_sdt=0%2C5&q=%28%22Peritumor%22%7C+%22Peritumoral%22%2B%22tissue%22%7C+%22juxtatumoral%22%2B%22tissue%22%7C+%22Non+malignant+Adjacent%22%2B%22tissue%22%7C+%22Tumor+Adjacent%22%2B%22tissue%22%7C+%22Tumor+macroenvironment%22%7C+%E2%80%9CExtratumoral+microenvironment%E2%80%9D%7C+%22Adjacent+non-neoplastic%22%7C+%22Non-tumor%22%7C+%22Cancer-adjacent%22%7C+%22Adjacent+to+Tumor%22%7C+%22Tumor-Surrounding%22%7C+%22Extratumoral+microenvironment%22%7C+%22Normal+adjacent%22%7C+%22Adjacent+normal+%22%7C+%22Neighboring+Normal%22%7C+%22Proximal+Normal%22%7C+%22Normal+tissue+adjacent%22%7C+%22Surrounding+Normal+Tissue%22%7C+%22Normal+appearing%22%7C+%22Histologically+Normal%22%7C+%22Morphologically+normal+tissue%22%7C+%22Healthy+Adjacent%22%7C+%22Adjacent+Healthy+%22%7C+%22Benign+Adjacent+%22%7C+%22Morphologically+normal+%22%7C+%22Adjacent+Benign+%22%29%2B%28%22Tumor%22%7C+%22Cancer%22%7C+%22Carcinoma%22%7C+%22Adenoma%22%7C+%22Sarcoma%22%29%2B%22tissue%22&btnG=) | 57000^b^ |

a| Query used for supplementary figure 1.
b| Google scholar query is not limited on title/abstract.
